# Case report: Delusional infestation in dementia with Lewy bodies

**DOI:** 10.3389/fpsyt.2022.1051067

**Published:** 2022-11-10

**Authors:** Daiki Taomoto, Hideki Kanemoto, Yuto Satake, Kenji Yoshiyama, Masao Iwase, Mamoru Hashimoto, Manabu Ikeda

**Affiliations:** ^1^Department of Psychiatry, Osaka University Graduate School of Medicine, Suita, Japan; ^2^Department of Neuropsychiatry, Kindai University Faculty of Medicine, Osakasayama, Japan

**Keywords:** Ekbom syndrome, delusional infestation, delusional parasitosis, tactile hallucination, dementia with Lewy bodies

## Abstract

**Background:**

Delusional infestation is characterized by delusions of being infested with parasites, vermin, or small insects and is frequently accompanied by tactile and visual hallucinations. Herein, we report two cases of dementia with Lewy bodies (DLB) with delusional infestation.

**Case presentation:**

Case 1 was an 83-year-old man. At the age of 75, he began to show symptoms of rapid eye movement sleep behavior disorder. At the age of 83, he began to complain of visual hallucinations of people and delusional infestation with tactile and visual hallucinations of insects, resulting in the use of insecticides for non-existent insects. He also complained of mild amnesia and was admitted to our psychiatric ward for evaluation and treatment. After admission, the delusional infestation disappeared without any new medication. Based on our examinations, he was diagnosed with probable DLB with delusional infestation. He was treated with 5 mg/day of donepezil hydrochloride; his visual and tactile hallucinations disappeared, and the delusional infestation had not recurred at the 1-year follow-up. Case 2 was a 69-year-old woman. At the age of 60, she underwent clipping for subarachnoid hemorrhage (SAH). At the age of 65, she began to have visual hallucinations of people. At the age of 67, she began to complain of visual illusions in which she mistook lint for insects. At the age of 69, she developed delusional infestation and mild amnesia. She took various actions to get rid of these non-existent insects, including insecticide use, consulting an exterminator, and visiting several dermatologists. She eventually burnt her leg in an attempt to kill the non-existent insects. Based on our examinations, she was diagnosed with prodromal DLB in addition to SAH sequelae. We determined that her delusional infestation was caused by DLB rather than SAH sequelae based on the course of her symptoms. She was treated with a combination of 3 mg/day of donepezil hydrochloride and 12.5 mg/day of quetiapine. Thereafter, the delusional infestation partially improved, and she took no further action against non-existent insects.

**Conclusion:**

Delusional infestation may be caused by DLB. Acetylcholinesterase inhibitors (AChEI) may be effective for delusional infestation in DLB, although antipsychotics may also be needed in severe cases.

## Introduction

Delusional infestation, also known as delusional parasitosis or Ekbom syndrome, is characterized by delusions of being infested with parasites, vermin, or small insects (1), as well as tactile and visual hallucinations (2). Although patients with this syndrome are sometimes diagnosed with delusional disorders, schizophrenia, depression, and dementia, the neurobiological mechanisms are not fully understood (3).

Dementia with Lewy bodies (DLB) is the second most common type of neurodegenerative dementia in older adults after Alzheimer’s dementia (4). Hallucinations and delusions are more common in patients with DLB than in patients with Alzheimer’s dementia (5). Psychosis in older people are frequently developed in prodromal stage and early stage of dementia (6). However, there are only a few reports on delusional infestation in DLB (7–9). Herein, we report our experience with two patients with DLB and delusional infestation who were both safely treated with medication.

## Case presentation

### Case 1

**FIGURE 1:**
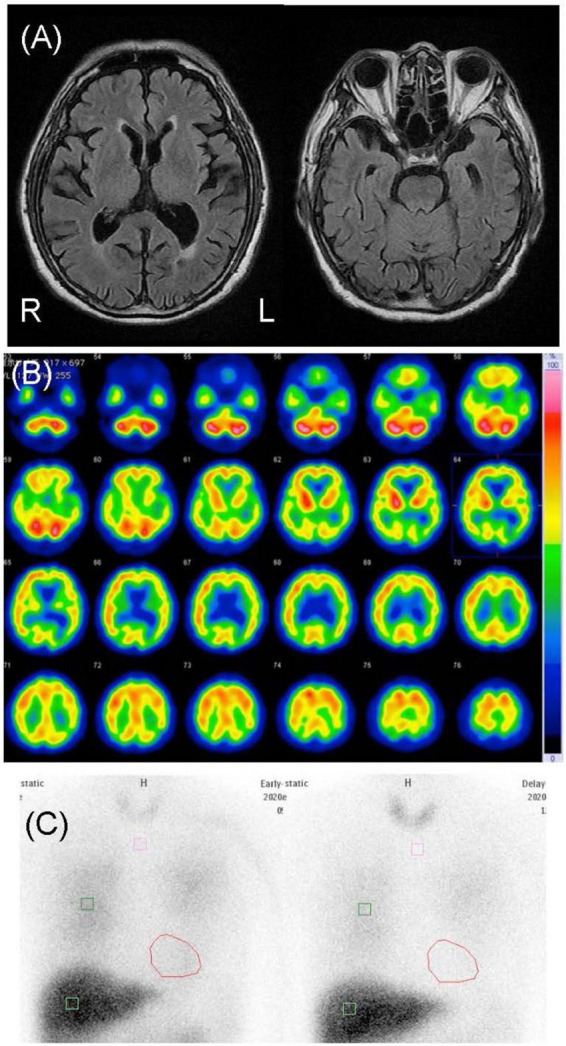
Neuroimages of case 1. **(A)** Cranial magnetic resonance images revealed diffuse mild atrophy including the bilateral hippocampus. **(B)** N-isopropyl-p-[^123^I] iodoamphetamine single-photon emission computed tomography revealed hypoperfusion in the bilateral occipital and parietal lobes. **(C)** Cardiac [^123^I]-metaiodobenzylguanidine scintigraphy revealed heart-to-mediastinum ratios of 1.55 and 1.26 in the early and delayed phases, respectively.

This patient was an 83-year-old man with a junior high school education who worked in the printing industry until the age of 73. His past medical history included hypertension, hyperuricemia, and inguinal hernia, with no history of substance abuse or heavy drinking or a family history of neuropsychiatric illness. At the age of 75, he began moving his limbs while sleeping and falling out of bed, which was thought to be caused by rapid eye movement sleep behavior disorder. At the age of 83, he began to complain of visual hallucinations of people and delusional infestation with tactile and visual hallucinations of insects. He used insecticides for non-existent insects, although he had some insight into his own symptoms. He stated, “I saw some insects that were 5 mm to 1 cm in size. I couldn’t touch them because they moved when I tried to touch them,” “The insects crawled on my body and bit me. They sometimes come out of my anus,” and “I think that this is a strange experience.” When we pointed out that there were no insects, he agreed with us” He also complained of mild amnesia and was admitted to our psychiatric ward for evaluation and treatment. After admission, his delusional infestation disappeared without any new medication; however, he developed visual and tactile hallucinations of people. He said “I saw a man last night. He painted me with a slimy thing,” and “A person in white clothes like a nurse touched me.” The patient had no visible skin abnormalities, and no neurological abnormalities, including parkinsonism, were observed. His Mini-Mental State Examination (MMSE) scores fluctuated from 18 to 23, reflecting his fluctuating cognition level. Blood and cerebrospinal fluid test results were normal. An electroencephalogram showed unremarkable findings. Cranial magnetic resonance imaging (MRI) revealed diffuse very mild atrophy including the bilateral hippocampus (Figure 1). N-isopropyl-p-[^123^I] iodoamphetamine (^123^I-IMP) single-photon emission computed tomography (SPECT) revealed hypoperfusion in the bilateral occipital and parietal lobes (Figure 1). Furthermore, cardiac ^123^I-metaiodobenzylguanidine scintigraphy revealed reduced myocardial uptake (heart-to-mediastinum ratio = early: 1.55; delayed: 1.26; Figure 1). Considering the results of all examinations, the patient was diagnosed with dementia because he showed progressive cognitive decline that interfered with his daily activities. Moreover, the patient had three core clinical features (rapid eye movement sleep behavior disorder, fluctuating cognition level, visual hallucinations) and one indicative biomarker (abnormal cardiac ^123^I-metaiodobenzylguanidine scintigraphy) and was therefore diagnosed with probable DLB (10) with delusional infestation. He was administered 5 mg/day of donepezil hydrochloride; his tactile and visual hallucinations disappeared, and the delusional infestation did not recur. Therefore, we did not increase the donepezil hydrochloride to the recommended dose of 10 mg/day. At the follow-up examination 1 year later, his symptoms had not recurred and his MMSE score remained unchanged (11), as did his ability to perform activities of daily living. His family members expressed understanding of the cause of and required treatment for delusional infestation.

### Case 2

**FIGURE 2:**
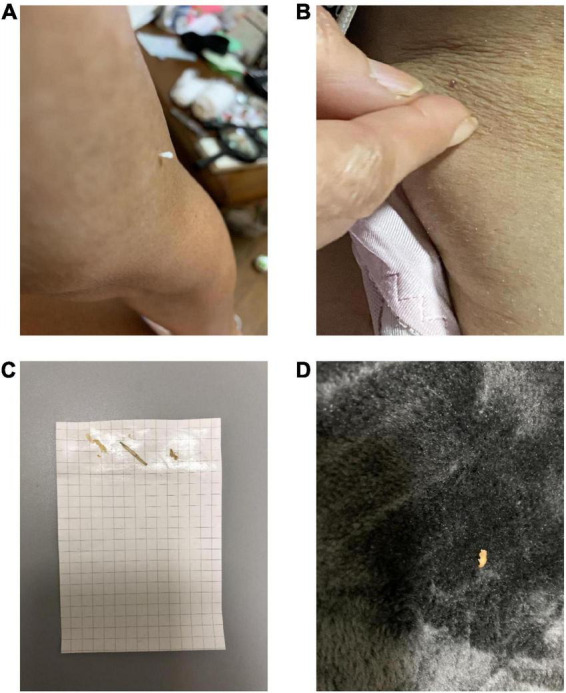
The patient in case 2 showed us pictures of lint that she thought looked like insects, which is known as the “specimen sign” or “matchbox sign”. **(A,B)** Picture of an object on her skin. **(C)** She pasted the object onto a paper and **(D)** found the object on the carpet.

**FIGURE 3:**
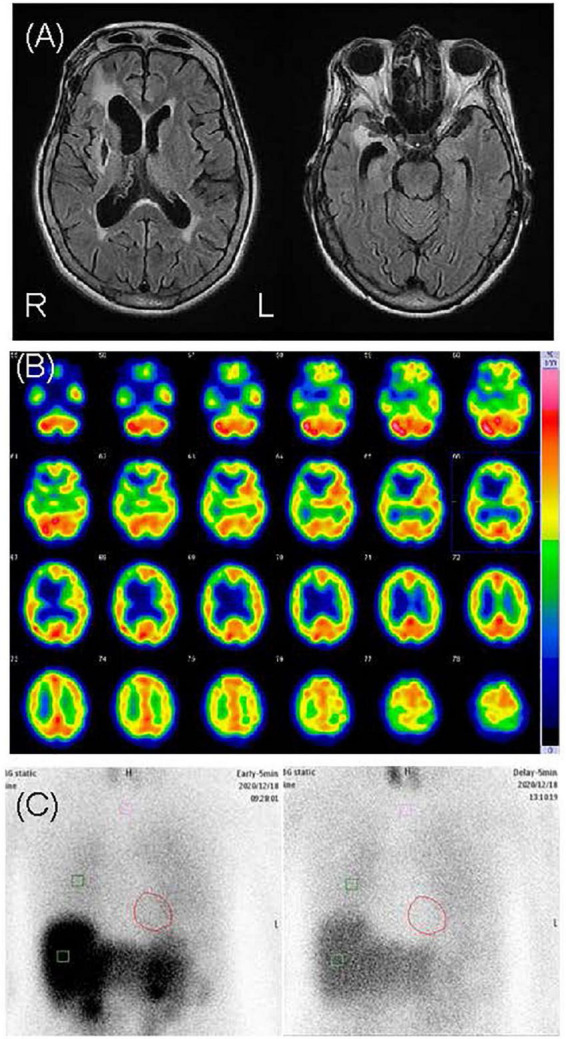
Neuroimages of case 2. **(A)** Cranial magnetic resonance revealed right dominant bilateral diffuse atrophy and right putamen chronic hemorrhage. **(B)** N-isopropyl-p-[^123^I] iodoamphetamine single-photon emission computed tomography revealed hypoperfusion in the right basal ganglia and the frontal, temporal, and parietal lobes. **(C)** Cardiac [^123^I]-metaiodobenzylguanidine scintigraphy revealed heart-to-mediastinum ratios of 1.91 and 1.32 in the early and delayed phases, respectively.

This patient was a 69-year-old woman who dropped out of high school and worked as a fashion model. Her past medical history included subarachnoid hemorrhage (SAH), symptomatic epilepsy following SAH, and hypertension. She had no family history of neuropsychiatric illness, nor did she have a history of substance abuse or heavy drinking. At the age of 60, she underwent clipping for SAH. At the age of 65, she began to have frequent visual hallucinations of people, although she had previously experienced visual hallucinations temporarily for a short period after the SAH clipping. She remembered the visual hallucinations she experienced after the clipping well, and these temporary visual hallucinations were not considered postoperative delirium. At the age of 67, she began to complain of visual illusions in which she mistook lint for insects. At the age of 69, she developed delusional infestation and saw insects crawling on her body. She had no insight into her symptoms and took various actions to get rid of these non-existent insects, including insecticide use, consulting an exterminator, moving to a different house, and visiting several dermatologists. Despite all evidence to the contrary, she had a fixed belief of being infested with insects. She also complained of mild amnesia. Her partner brought her to our psychiatric clinic for evaluation and treatment. Her delusion was severe. She stated, “There are white insects in my body. They come out of my skin,” “Sometimes I can see crawling insects. But I can’t see insects when they crawl in my clothes,” and “They crawled on the skin of my neck, trunk, and limbs. I felt tingling.” She showed us pictures of lint on her limbs, claiming they were pictures of insects (Figure 2). Her partner said, “When I pointed out that there were no insects, she disagreed with me and insisted that they were present.” Neurological examination revealed mild paralysis and sensory deficits in the left upper and lower limbs as sequelae of SAH, although she did not have parkinsonism. Her MMSE scores ranged from 21 to 26, reflecting her fluctuating cognition level. The blood and cerebral spinal fluid test results showed no abnormalities. An electroencephalogram showed sharp waves in the right frontal area, which was considered an effect of SAH, with no obvious slow wave. Cranial MRI revealed an old hemorrhagic lesion in the right putamen and right dominant bilateral diffuse atrophy with ischemic changes secondary to SAH (Figure 3). ^123^I-IMP SPECT revealed hypoperfusion in the right basal ganglia and the frontal, temporal, and parietal lobes (Figure 3). Furthermore, cardiac ^123^I-metaiodobenzylguanidine scintigraphy revealed reduced myocardial uptake (heart-to-mediastinum ratio = early: 1.91; delayed: 1.32; Figure 3). Considering the results of all examinations, the patient was diagnosed with mild cognitive impairment because she showed progressive cognitive decline that did not to interfere with her daily activities. She had two clinical features (fluctuating cognition level, visual hallucinations) and one indicative biomarker (abnormal cardiac ^123^I metaiodobenzylguanidine scintigraphy) and was therefore diagnosed with prodromal DLB (12) in addition to SAH sequelae. We determined that her delusional infestation was caused by DLB rather than SAH sequelae based on the course of her symptoms. Seven days after the initiation of 3 mg/day of donepezil hydrochloride, she visited another hospital because she had burnt her own leg in an attempt to kill the non-existent insects. When she visited our outpatient clinic after treatment for her burns, we determined that her behavior was not due to drug-induced impulsivity related to donepezil hydrochloride, but rather to behavioralization associated with the delusional infestation with visual hallucinations. Therefore, donepezil hydrochloride was increased to 10 mg/day with the aim of improving her symptoms. After donepezil hydrochloride was increased to 10 mg/day, her visual hallucinations of people improved. However, the delusional infestation with visual illusions of insects continued. Therefore, she was admitted to our hospital for treatment of delusional infestation. She persistently complained of delusional infestation and repeatedly showed us lint that looked like insects to her. She was prescribed 12.5 mg/day of quetiapine, and her delusional infestation partially improved. Donepezil hydrochloride was reduced to 3 mg/day because of frequent urination. Ultimately, she was treated with a combination of 3 mg/day of donepezil hydrochloride and 12.5 mg/day of quetiapine. The delusional infestation partially improved, and she took no further action against non-existent insects until a year later at our last follow-up. At the follow-up examination 1 year later, her MMSE score was 25, which was not significantly difference from 1 year earlier, and her impairment regarding activities of daily living remained unchanged. Her partner expressed understanding regarding the cause of and treatment for delusional infestation, and stated that he had stopped to point out the absence of the insects because he found that this was ineffective.

## Discussion

We report our experience with two cases of DLB with delusional infestation. Both patients complained of insects coming out of their bodies and used insecticides for non-existent insects. Case 1 had some insight into his symptoms. After he was admitted, his delusional infestation disappeared without any new medication; however, he developed tactile hallucinations of people. The content of the tactile hallucinations depended on the content of his visual hallucinations. He did not take any other action against the non-existent insects other than insecticide use. On the contrary, Case 2 had no insight into her symptoms. After she was admitted, her delusional infestation continued, and she had no tactile hallucinations other than those of insects. Her tactile hallucinations and delusional infestation sometimes appeared in the absence of visual hallucinations. She took various actions against these non-existent insects, including consulting an exterminator, moving to a different house, visiting several dermatologists, and insecticide use. She eventually burnt her leg in attempt to remove the non-existent insects, similar to the patient reported by Von Ekbom (1). Case 2 showed us pictures of lint that she thought looked like insects (Figure 2), which is known as the “specimen sign” (13) or “matchbox sign” (14). The differences in delusional infestation and tactile hallucinations between these two patients indicate that the delusional infestation in case 2 was more severe than that in case 1. Differences in the severity of delusional infestation may affect the patient’s treatment response.

Case 1 was diagnosed with probable DLB (10) and case 2 was diagnosed with prodromal DLB (12). Both patients had visual hallucinations, which is one of the core clinical features of DLB. Among four prior cases of delusional infestation diagnosed with DLB, two patients had visual hallucinations (7–9). Even in cases of suspected dementia in which a diagnosis of DLB had not been made, the coexistence of complex visual hallucinations and delusional infestation have been reported (15–17). The patient reported by Kanazawa and Hata was suspected of having Alzheimer’s dementia (15), and the case was reported before the publication of the first criteria for DLB (18). Although the patient reported by Rocha et al. showed cognitive impairment, the diagnosis was unclear because the patient refused detailed examinations and evaluation and was also limited by visual deficits and hypoacusis (16). Another case reported by Hirakawa et al. was suspected of having DLB, and donepezil hydrochloride was prescribed (17). Delusional infestation occasionally accompanies visual hallucinations (13), possibly suggesting the presence of a certain number of cases of undiagnosed DLB among patients with dementia and delusional infestation. In fact, if the indicative biomarkers were not available, Case 2 might have been diagnosed with delusional infestation caused by SAH.

Although little is known about the pathophysiology of delusional infestation (3), a promising hypothesis involves deterioration of the striatal dopamine transporter function (19), which is a hallmark of DLB. If this hypothesis is correct, it is possible that some patients with delusional infestation without dementia may also have the psychiatric-onset type of prodromal DLB (12). We recently compared the characteristics of patients with very-late-onset schizophrenia-like psychosis with and without positive results for the indicative biomarkers of DLB and the prevalence of visual hallucination was higher in patients with very-late-onset schizophrenia-like psychosis and suspected prodromal DLB (20). Visual hallucinations may be a symptom suggestive of DLB, even in older patients with delusional infestation.

There is limited existing evidence related to brain images in cases of delusional infestation, with only a few case reports and one case series available in the literature (21). Huber et al. reported a possible relationship between the striatum, particularly the putamen, and delusional infestation (21). Other case reports suggest that various regions of brain damage affect delusional infestation, such as damage in the right temporo-parietal (22, 23), right temporo-occipital (11, 24), left temporo-occipital (25), and right frontal lobes (22). In case 1, cranial MRI revealed diffuse very mild atrophy, including the bilateral hippocampus, and a ^123^I-IMP SPECT revealed hypoperfusion in the bilateral occipital and parietal lobes (Figure 1). These results are consistent with the characteristics of DLB, and we could not find an apparent difference in brain images between case 1 and DLB patients without delusional infestation. In case 2, cranial MRI revealed an old hemorrhagic lesion in the right putamen, right dominant bilateral diffuse atrophy, and ischemic changes secondary to SAH, while ^123^I-IMP SPECT revealed hypoperfusion in the right basal ganglia and the frontal, temporal, and parietal lobes (Figure 3). This patient’s brain lesions in the right putamen and the frontal, temporal, and parietal lobes were consistent with those in past reports. Therefore, we considered that SAH might have partially affected her severe delusional infestation. However, we determined that her delusional infestation was mainly caused by DLB rather than SAH sequelae based on the course of her symptoms. Therefore, we considered that case 2 had increased vulnerability for delusional infestation after SAH, and that her delusional infestation appeared after the onset of DLB.

After admission, the patient in case 1 had no further complaints of delusional infestation. His visual and tactile hallucinations, which frequently accompanied his delusional infestation, disappeared with donepezil hydrochloride administration, and his delusional infestation had not recurred at the 1-year follow-up with continued donepezil hydrochloride use, even after discharge. Although case 2 continued to complain of delusional infestation with visual illusions, severity of the delusional hallucination was apparently improved, and her visual hallucinations disappeared after the initiation of donepezil hydrochloride. Moreover, her delusional infestation further improved after adding quetiapine to her medication regimen. Mori et al. reported that donepezil hydrochloride was effective for visual hallucinations in patients with DLB (26). In cases 1 and 2, donepezil hydrochloride was effective for reducing the frequency of or eliminating visual hallucinations. However, the efficacy of donepezil hydrochloride for delusional infestation in patients with DLB differed between cases 1 and 2. There have been some case reports of treatment for delusional infestation in DLB. Magierski et al. reported two cases of delusional infestation in patients with DLB who were treated with a combination of acetylcholinesterase inhibitor (AChEI) therapy and 50 mg/day of quetiapine or 2.5–5 mg/day of olanzapine (7). Ochiai et al. reported a patient with delusional infestation and depressive symptoms with DLB who was treated with a combination of 5 mg/day of donepezil hydrochloride and 1.5 mg/day of aripiprazole, with temporary administration of mirtazapine (8). de Mendonça et al. reported a DLB patient with delusional infestation and depressive symptoms who was treated with a combination of rivastigmine and citalopram (9). All patients in all cases, including ours, were safely treated with medication. According to the past cases and our case 2, antipsychotics may be necessary for partial or full remission of delusional infestation in patients with DLB. However, de Mendonça et al.’s patient and our case 1 were successfully treated without antipsychotics. Recent reviews for the treatment of delusional infestation recommend antipsychotics but do not include AChEI as a treatment option (27, 28). However, DLB has severe sensitivity to antipsychotics, which may cause worsening cognition, sedation, acute onset parkinsonism, and symptoms resembling neuroleptic malignant syndrome (29). Thereafter, it may be more beneficial to use AChEI initially and antipsychotics only when AChEI is ineffective.

Case 2 burnt her leg in an attempt to kill non-existent insects after the initiation of donepezil hydrochloride. Carrasco et al. reported that the frequency of agitation was 1.1% among patients with Alzheimer’s dementia who were prescribed donepezil hydrochloride (30). After our patient burnt her leg, she continued with an increased dose of donepezil hydrochloride with no further agitation or impulsive behavior. Therefore, we cannot conclude that her impulsive behavior, that is burning her own legs, was a side effect of donepezil hydrochloride. However, in patients with DLB with delusional infestation such behavior may occur due to increased impulsivity induced by donepezil hydrochloride, which should therefore be administered with caution.

In summary, delusional infestation is rare and can occur in patients with DLB. It is often accompanied by visual hallucinations related to the delusion. Our findings indicate that it may be beneficial to use AChEI as priority and antipsychotics only when AChEI is ineffective for delusional infestation in patients with DLB. Further studies on the underlying pathophysiology and treatment of delusional infestation in patients with DLB are required.

## Data availability statement

The original contributions presented in the study are included in the article/supplementary material, further inquiries can be directed to the corresponding author.

## Ethics statement

Ethical review and approval was not required for the study on human participants in accordance with the local legislation and institutional requirements. The patients/participants provided their written informed consent to participate in this study.

## Author contributions

DT conducted the neuropsychological assessments of patients, collected the data, and wrote the initial draft of the manuscript. MIk conducted the outpatient treatments. HK, YS, KY, MIw, MH, and MIk offered treatment advice and participated in the discussion of the results. All authors contributed to the manuscript and approved the final version for submission.
